# Neural Functioning in Late-Life Depression: An Activation Likelihood Estimation Meta-Analysis

**DOI:** 10.3390/geriatrics9040087

**Published:** 2024-06-25

**Authors:** Antonio Del Casale, Serena Mancino, Jan Francesco Arena, Grazia Fernanda Spitoni, Elisa Campanini, Barbara Adriani, Laura Tafaro, Alessandro Alcibiade, Giacomo Ciocca, Andrea Romano, Alessandro Bozzao, Stefano Ferracuti

**Affiliations:** 1 Department of Dynamic and Clinical Psychology and Health Studies, Faculty of Medicine and Psychology, Sapienza University of Rome, 00185 Rome, Italy; 2 Unit of Psychiatry, Emergency and Admissions Department, ‘Sant’Andrea’ University Hospital, 00189 Rome, Italy; 3 Department of Neuroscience, Mental Health and Sensory Organs (NESMOS), Faculty of Medicine and Psychology, Sapienza University, 00189 Rome, Italy; 4 Department of Clinical and Molecular Medicine, Sapienza University, 00189 Rome, Italy; laura.tafaro@uniroma1.it; 5 Unit of Internal Medicine, ‘Sant’Andrea’ University Hospital, 00189 Rome, Italy; 6 Marina Militare Italiana (Italian Navy), Ministry of Defence, Piazza della Marina, 4, 00196 Rome, Italy; 7 Unit of Neuroradiology, Department of Diagnostic Sciences, ‘Sant’Andrea’ University Hospital, 00189 Rome, Italy; 8 Department of Human Neuroscience, Faculty of Medicine and Dentistry, Sapienza University of Rome, 00185 Rome, Italy; 9 Unit of Risk Management, ‘Sant’Andrea’ University Hospital, 00189 Rome, Italy

**Keywords:** depressive disorder, late onset disorders, functional neuroimaging, geriatric psychiatry, superior temporal gyrus, medial frontal gyrus

## Abstract

Late-life depression (LLD) is a relatively common and debilitating mental disorder, also associated with cognitive dysfunctions and an increased risk of mortality. Considering the growing elderly population worldwide, LLD is increasingly emerging as a significant public health issue, also due to the rise in direct and indirect costs borne by healthcare systems. Understanding the neuroanatomical and neurofunctional correlates of LLD is crucial for developing more targeted and effective interventions, both from a preventive and therapeutic standpoint. This ALE meta-analysis aims to evaluate the involvement of specific neurofunctional changes in the neurophysiopathology of LLD by analysing functional neuroimaging studies conducted on patients with LLD compared to healthy subjects (HCs). We included 19 studies conducted on 844 subjects, divided into 439 patients with LLD and 405 HCs. Patients with LLD, compared to HCs, showed significant hypoactivation of the right superior and medial frontal gyri (Brodmann areas (Bas) 8, 9), left cingulate cortex (BA 24), left putamen, and left caudate body. The same patients exhibited significant hyperactivation of the left superior temporal gyrus (BA 42), left inferior frontal gyrus (BA 45), right anterior cingulate cortex (BA 24), right cerebellar culmen, and left cerebellar declive. In summary, we found significant changes in activation patterns and brain functioning in areas encompassed in the cortico–limbic–striatal network in LLD. Furthermore, our results suggest a potential role for areas within the cortico–striatal–cerebellar network in the neurophysiopathology of LLD.

## 1. Introduction

Late-life depression (LLD) is a relatively common and debilitating mental disorder, also associated with cognitive dysfunctions that may persist after effective treatment [1,2] and with increased risk of mortality [3,4]. The prevalence of depressive symptoms is around 17% in individuals 75 years old and older and 19% in individuals 50 years old and older [2]. In the elderly population, late-life depression poses a significant health concern, often linked to comorbidity, reduced functioning, high healthcare utilization, and increased mortality, including suicide risk. The DSM-5 defines major depressive disorder (MDD) by the presence of depressed mood or loss of interest, along with several associated symptoms such as changes in appetite, sleep, energy, concentration, feelings of guilt, and recurrent thoughts of death. Diagnosis requires impairment in social, occupational, or other areas of functioning, with symptoms persisting for at least two weeks.

There is a debate on the onset age of LLD in the literature. Depressive syndromes have shown neurobiological differences depending on the age of first onset. However, when previous authors have looked for differences between early-onset LLD (EOD) and late-onset LLD (LOD), they have not always been successful in identifying significant clinical differences [5]. LLD is usually referred to as MDD occurring in people aged 60 or older [6]. However, relevant differences in this age cut-off distinguishing between EOD and LOD can be found across the literature, with studies most commonly reporting EOD/LOD cut-offs at 60, 50, and 65 years old [5,6]. Some authors found that by adopting the definition of LOD as MDD occurring after the age of 65 years, a different clinical syndrome with significant differences in memory dysfunction and liability to movement disorders can be identified, highlighting how the research is still in the middle of its development [6]. In this study we have considered LLD as occurring in people aged 60 or older, i.e., the most frequent cut-off in the functional neuroimaging literature on this topic.

Addressing late-onset depression necessitates the collaboration of an interprofessional team to ensure optimal patient care. Given the growing global elderly population, LLD has emerged as a significant public health issue, also because the persistence of depressive symptoms could be a burden to society by growth of mean annual direct costs [7].

Some evidence has also linked LLD to an increased risk of Alzheimer’s disease, suggesting potential common pathophysiological aspects between the two disorders [8,9]. For example, amyloid deposition has been correlated with the manifestation of depression, albeit with conflicting results [10]. Other factors that may be implicated in the differential diagnosis in the elderly patient include neurodegeneration and cerebrovascular disease, which may in turn be involved in the pathophysiology of depressive syndromes, cognitive decline, and the onset of clinically relevant cognitive impairment [11,12].

The protective nature of physical activity has been consistently acknowledged. The most prevalent risk factors include chronic diseases, difficulty initiating sleep, mobility loss, impairment in performing daily activities, as well as vision impairment. Alcohol consumption, smoking behaviour, and other psychosocial issues could also be considered as risk factors [13]. Additionally, further biological aspects may be correlated with LLD onset [6,14].

Exploring the intricate connections among neuroanatomy, neurophysiology, and LLD is crucial in the pursuit of more precise and impactful diagnostics and interventions. Investigating the underlying neural mechanisms involved in LLD provides a deeper perspective that may unlock new insights to enhance therapeutic approaches and, consequently, improve the lives of people grappling with this challenging condition.

To date, numerous studies have investigated the phenomenon. At the morphometric level, several studies have highlighted structural brain changes in LLD. Regions most affected by grey matter alterations include the temporal, prefrontal, and parietal cortices, as well as the hippocampus, thalamus, and putamen, albeit with significant discrepancies [15]. Overall, structural alterations in areas encompassed in the fronto–striatal–limbic network may be implicated in the neurophysiopathology of LLD [16].

Functional neuroimaging studies have identified various anomalies, including changes in activation and connectivity in various brain structures, including the superior and inferior frontal gyri, praecuneus, precentral gyrus, cingulate gyrus, parahippocampal cortex, cerebellum, or putamen [17,18,19,20,21]. Also, regarding the functional neural correlates of LLD, evidence appears sometimes inconsistent, as underscored by a recent meta-analysis that failed to identify structural and functional changes in LLD [15]. However, these negative results may also be due to the diversity of methodologies of included studies and the types of patients included in studies subjected to meta-analysis.

The data briefly reported so far suggests an inconsistency both in the evidence itself and in the methods employed for the investigations. Additionally, to our knowledge, there are no meta-analyses in the literature focused exclusively on neurofunctional studies conducted on patients with LLD. Although individual studies have shown variable results regarding structural and functional brain changes in LLD, a comprehensive understanding of the involvement of specific brain regions within a network framework may provide valuable insights into the neurobiological mechanisms underlying LLD.

Based on our observations, this study presents an Activation Likelihood Estimation (ALE) meta-analysis aimed at analyzing specific brain changes by examining functional neuroimaging studies conducted on patients with LLD compared to healthy subjects.

The main objective of this study is twofold. Firstly, we aim to identify consistent evidence of neurofunctional alterations characteristic of LLD. By doing so, we hope to enhance our understanding of the underlying neurobiological mechanisms contributing to LLD. Secondly, we aim to lay the groundwork for potential diagnostic advancements and personalized treatment innovations.

To achieve this, we will utilize the ALE method, as it has become the standard for functional neuroimaging meta-analyses. The ALE method models the three-dimensional coordinates (obtained from reported activations in a standard space) as the center of a three-dimensional Gaussian distribution, thereby expanding the potential pool of studies eligible for meta-analysis. Additionally, it allows for whole-brain analyses with corrections for multiple comparisons [22,23,24].

Specifically, we will employ this methodology to test the hypothesis that alterations in activation patterns and cerebral functioning within cortico–limbic–striatal areas may contribute to the pathophysiology of LLD.

## 2. Methods

A search was conducted on 9 April 2024 using the international scientific database PubMed (http://www.pubmed.gov, accessed on 2 May 2024) to identify functional neuroimaging studies that analysed brain functional changes in patients affected by LLD compared to healthy controls (HCs). We followed the methods of the Preferred Reporting Items for Systematic Reviews and Meta-Analyses (PRISMA Statement) [25].

We searched the database PubMed using the “title/abstract” filter with the search string ‘depress* AND (elderly OR old-age OR late life OR geriatr*) AND (single photon emission computed tomography OR positron emission tomography OR functional magnetic resonance OR fMRI OR PET OR SPECT) NOT review [PT]’ and excluding the terms “Alzheimer”, “stroke”, “migraine”, and “pet therapy”. Additionally, we searched for further studies by examining the bibliographies of relevant articles in the first step or through the “related article” function of the PubMed database and bibliographies of reviews on the topic.

We included articles describing functional neuroimaging studies on patients diagnosed with LLD, considering LLD as MMD occurring in people aged 60 or older [6]. We excluded studies that used neuroimaging techniques other than functional magnetic resonance imaging (fMRI), positron emission tomography (PET), and single-photon emission computed tomography (SPECT); studies exclusively focused on functional connectivity between different brain areas; correlation studies; studies without comparisons with HCs; articles not reporting functional neuroimaging results with coordinates; and articles focused on other diagnoses. Additionally, we excluded studies with participants of different ages not corresponding to LLD, studies on treatment response, case reports, studies conducted on patients with severe organic comorbidities, brain stimulation studies, animal studies, neuropharmacological studies, neurophysiological studies, meta-analyses, and study protocols.

**Figure 1:**
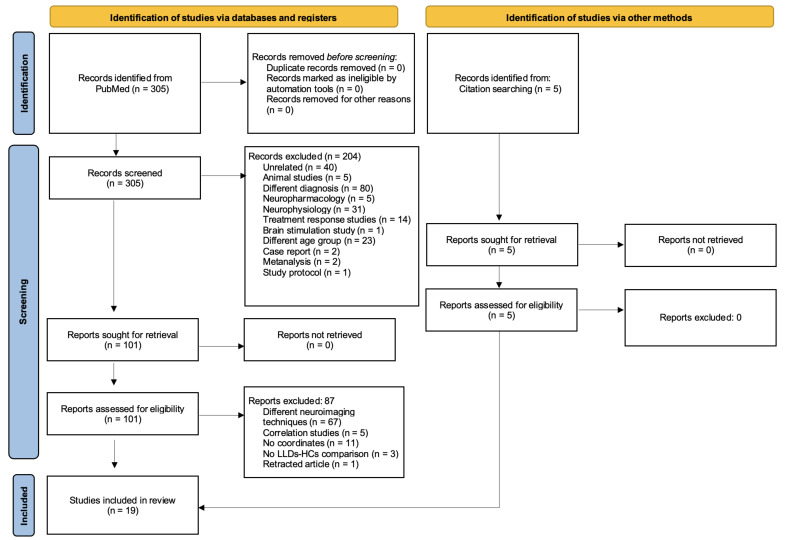
Search strategy (PRISMA 2020 flow diagram).

Based on these criteria, we analysed 305 studies, from which we excluded 281 studies, and included 5 other studies from the bibliographic search, finally including 19 studies (14 from database and registers and 5 from other methods) published before 2023 [17,19,20,26,27,28,29,30,31,32,33,34,35,36,37,38,39,40,41]. We report the search flow diagram in Figure 1.

*Statistical analyses*. We created a database with the coordinates of the studies referring to the contrast LLD versus healthy state by cluster of significant activation differences corrected for false positives. Then, we performed a single analysis of the data set with the BrainMap GingerALE 3.0.2 software (http://www.brainmap.org/ale/, accessed on 2 May 2024). The included experiments focused on emotional tasks, attentional-memory based tasks, verbal fluency tasks, and resting state cerebral blood oxygen level-dependent (BOLD) activations (regional homogeneity, low-frequency amplitude of the signal, percentage of amplitude fluctuation) and cerebral metabolism. This made it possible to assess changes in brain activations under different conditions in patients with LLD compared to HCs during task execution or in the resting state condition.

Data processing and statistical analysis: the coordinates in the Talairach space have been converted to the Montreal Neurological Institute (MNI) space with the GingerALE converting tool, so that all coordinates in this study are MNI. The meta-analyses were based on the ALE method, using the GingerALE 3.0.2 algorithm (http://www.brainmap.org/ale, accessed on 2 May 2024) [22,24].

Considering that all the coordinates in our database were just corrected for false positive in the original studies, we conducted a first analysis using the ALE method with uncorrected *p* values < 0.0001. Then, to obtain a further correction for false-positive results, we conducted another analysis using the corrected threshold for cluster-level inference, with an uncorrected *p* value of 0.0001 as the cluster formation threshold, a *p* value of 0.05 for cluster-level inference, and a threshold permutation value of 250. The images obtained were visualized using the Mango software (http://ric.uthscsa.edu/mango/, accessed on 2 May 2024) and superimposed on an anatomical model.

## 3. Results

Global characteristics of the enrolled studies: the included studies were conducted on 844 subjects, divided into 439 patients with LLD (148 men, 270 women, 20 with not specified gender; weighted average age: 64.71 years, SD = 6.31) and 405 HCs (153 men, 235 women, 17 with not specified gender; weighted average age: 69.01 years, SD = 6.02). Focusing on the drug treatments, 196 patients were not receiving medications at the time of the study, while 243 were on medications, predominantly comprising selective serotonin reuptake inhibitors (SSRIs) or serotonin and norepinephrine reuptake inhibitors (SNRIs) (12% of prescriptions), tricyclic or other antidepressants (3.9%), benzodiazepines (4.8%), and unspecified drugs (36.44%). Other treatments including antiepileptics, lithium, and other drugs were prescribed in less than 2% of cases. We summarized the sociodemographic and clinical characteristics of the study samples in Table 1.

*Between-group analysis*. The LLD < HCs contrasts included 14 experiments with 75 reported activation foci on 647 subjects. Patients with LLD, compared to HCs, showed significant hypofunctioning of the right superior and medial frontal gyri (Brodmann areas [BAs] 8, 9), left cingulate cortex (BA 24), left putamen, and left caudate body (*p* < 0.0001). The LLD > HCs contrasts included 14 experiments with 76 reported activation foci on 595 subjects. Patients with LLD, compared to HCs, showed significant hyperfunctioning of the left superior temporal gyrus (STG) (BA 42), left inferior frontal gyrus (BA 45), right anterior cingulate cortex (ACC) (BA 24), right cerebellar culmen, and left cerebellar declive (*p* < 0.0001) (Table 2).

**Figure 2:**
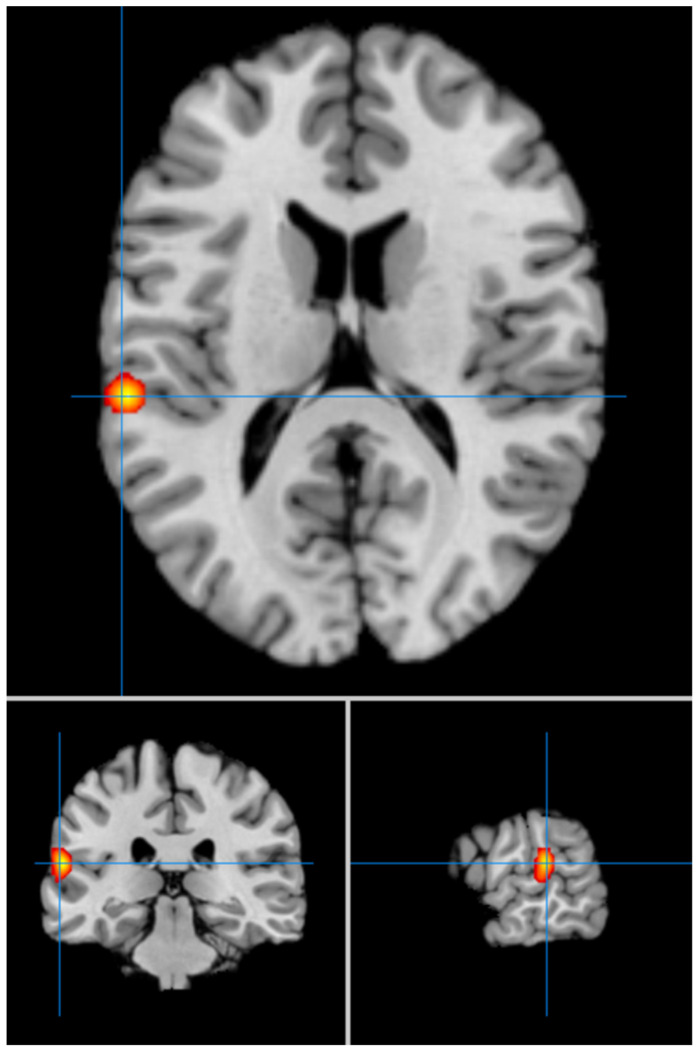
Significant hyperfunctioning of the left superior temporal gyrus (MNI: −60, −30, 14) in LLD (FWE corrected).

**Figure 3:**
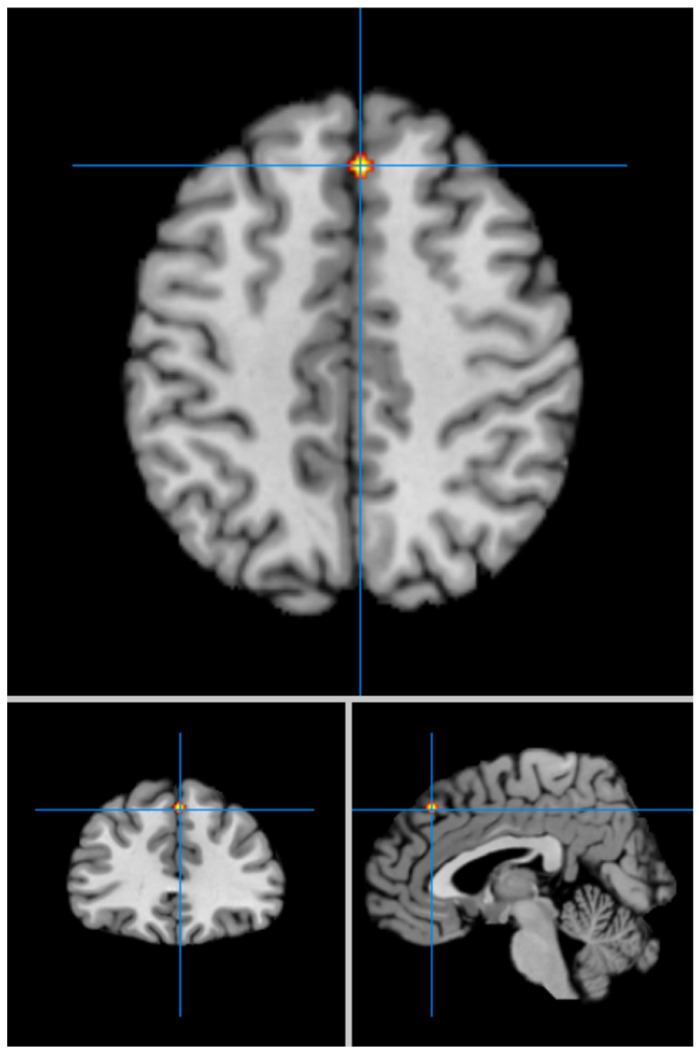
Significant hypofunctioning of the right medial/superior frontal gyri (MNI: 4, 32, 44) in LLD (FWE corrected).

Our second analysis with cluster-level correction showed in LLD vs. HCs significant hyperfunctioning of the left superior temporal gyrus (BA 42) (MNI coordinates x = −60; y = −30; z = 14) (FWE *p* < 0.05) (Figure 2), and significant hypofunctioning of the right medial frontal gyrus (BA 8) (MNI coordinates x = 4; y = 32; z = 44) (Figure 3). We summarize the results of our meta-analysis in Table 2.

## 4. Discussion

This meta-analysis confirmed our primary hypothesis, revealing significant changes in activation patterns and brain functioning within cortico–limbic–striatal areas associated with LD diagnosis compared to health status. Consistent with other studies, these alterations encompass both hyperactivation and hypoactivation of specific areas. Furthermore, the findings underscored the involvement of the cortico–striatal–cerebellar network in contributing to the motor, cognitive, and affective symptoms observed in LLD.

### 4.1. Cortical Areas

The first most important finding of our meta-analysis is the hypofunctioning of the right medial/superior frontal gyri (BA 8) in LLD compared to HCs. Traditionally known as the “frontal eye field”, BA 8 exhibits diverse functional involvement spanning motor, language, executive functions, memory, and attention [42,43,44,45,46].

Some neuroimaging studies in the literature provide relevant insights into our findings concerning LLD. For instance, previous research has linked the right BA 8 to emotional processing and regulation, as evidenced in adults with remitted MDD compared to HCs [47]. Furthermore, reports indicate reduced regional cerebral blood flow (rCBF) in this area among patients with Alzheimer’s disease and comorbid depressive symptoms [48]. Moreover, the right BA 8 is implicated in a neural network involving the right premotor cortex (BA 6), right orbitofrontal cortex (BA 10), and left posterior cingulate gyrus (BA 31), which has been associated with apathy and impaired recognition of emotional faces in patients with Parkinson’s disease [49].

The other most important result of our meta-analysis is the hyperfunctioning of the left STG (BA 42) in LLD vs. HCs. This area is physiologically involved in auditory stimuli processing [50], visual speech perception [51], being integrated with other regions within the mirror neuron system network (MSN) and possibly involved in social cognition and analysis of biological vs. non biological motion [52]. Our results are consistent with a recent study showing that decreased activation of the left STG in response to negative emotional expression is positively correlated with increases in positive affectivity in patients with LLD after eight weeks of mindfulness-based cognitive therapy [53]. Another study demonstrated increased regional homogeneity in this area in LLD patients compared to HCs [19], further confirming the involvement of the left STG in the neuropathophysiology of LLD.

From a structural point of view, the surface area of this cortex has been significantly correlated with the severity of depressive symptoms in elderly patients with subcortical vascular mild cognitive impairment [54].

BAs 8 and 42 can be considered critical nodes in the auditory/language network and the sensorimotor network, as well as in the broader cognitive framework. Perceptual systems representing the sensory system, including auditory, visual, and sensorimotor networks, can be seen as a sublevel within the hierarchical organization of cognitive and executive processes, including those involved in emotion processing. The changes we have highlighted within the context of the auditory processing network (STG) and cognitive and motor processing (BA 8) may in turn be involved in the manifestation of cognitive and perceptual disorders, as well as affective issues, in patients with LLD, consistent with previous studies [19,55].

Another cortical area that has emerged is the left inferior frontal gyrus (BA 45) demonstrates hyperfunctioning in LLD compared to HCs. Broca’s area is centrally implicated in language and is also pivotal in other cognitive functions, including memory [56,57] and motor control [58,59], as well as mirror neuron-related functions [60]. In LLD, increased functional connectivity between the left inferior frontal gyrus pars triangularis and the left frontal eye fields, along with their connectivity with other brain regions, has been correlated with a reduction in depressive symptoms over 12 weeks of treatment with sertraline [61]. Patients with LLD also exhibit hyperactivation of BA 45 during the encoding of semantically related words, highlighting its critical role in memory consolidation, cognitive control, and controlled semantic/phonological retrieval and analysis [40]. Furthermore, from a structural perspective, concurrent atrophy of the left inferior frontal gyrus has been linked to depressive symptoms in elderly patients with severe small vessel disease [62], affirming the crucial involvement of both the structure and function of this area in the neuropathophysiology of depressive conditions in the elderly.

### 4.2. Limbic Areas

We demonstrated that the right ACC (BA 24) hyperfunctions in LLD compared to the healthy state. BA 24 is implicated in various cognitive, affective, and behavioural processes, as well as language initiation and suppression, and pain processing [63,64,65,66,67,68]. The ACC plays a significant role in the neuropathophysiology of anxiety and personality traits, exhibiting variations that may also be influenced by gender [69,70]. Our findings align with a study conducted by Smith and colleagues [71], wherein they demonstrated improvements in depressive symptoms alongside reduced glucose metabolism in the right ACC in patients with LLD. Furthermore, the severity of depression has been inversely correlated with the right anterior cingulate blood flow in adult unmedicated patients with depression [72], and medication-free patients with MDD showed increased rCBF in the right ACC in a recent meta-analysis on studies conducted in adults [73]. The severity of gastrointestinal symptoms in MDD was negatively correlated with choline-containing compounds to creatine in the right ACC [74]. In line with these functional evidence, structural neuroimaging studies in adults showed smaller grey matter volumes of the right ACC both in bipolar and unipolar depression [75], and lower fractional anisotropy, i.e., an index tissue organization in patients with LLD in the right ACC [76]. These aspects can be linked with evidence of neural developmental abnormalities (cortical gyrification) and potential influence of neuroinflammation (levels of interleukin-6 and interleukin-8) on right ACC function in MDD patients [77]. Disfunctions of this area in subjects with the s/s genotype of the SLC6A4 promoter region of the serotonin transporter (5-HTT) have been considered as an “overactive metabolic state”, possibly related to an increased susceptibility for developing an anxiety-depression spectrum disorder [78].

We also observed hypoactivation of the left ACC (BA 32) in LLD vs. HCs. This result is in line with some neuroimaging studies found in the literature. Pioneering PET studies have demonstrated a decrease in glucose utilization in the ACC of patients with depression [79,80]. Furthermore, dysfunction of the left ACC has been considered as a neural substrate of cognitive impairment, particularly lower speed of processing, in adult patients with MDD and insomnia symptoms [81]. Abnormal left ACC neural activity may play a key role in the pathophysiology of first episode MDD [82]. This area has been also involved in other MDD symptoms in adult patients, considering the negative correlation between its activation and the severity of apathy [83], its reduced spontaneous neural activity in melancholic vs. non melancholic MDD [84], abnormal choline metabolism in unmedicated MDD patients who had experienced childhood trauma [85], and changes in choline and N-acetylaspartate levels in MDD with cognitive dysfunctions [86]. Left ACC functional and structural integrity has been also related to the clinical outcome of MDD and treatment response [86,87,88].

Specifically, regarding elderly patients, ACC activation has been significantly reduced in LLD patients who have experienced multiple depressive episodes during a verbal fluency task [38], as well as in patients undergoing brain activation paradigms [89]. Therefore, the decreased functionality of the ACC highlighted by our meta-analysis is consistent with these previous studies, appearing as a significant neurofunctional correlate involved in the pathophysiology of depression.

Our meta-analysis showed different lateralization functional correlates in LDD as compared to the healthy state. While the bilateral ACC is involved in a range of cognitive and emotional functions, there is evidence on functional lateralization, with the right ACC being more strongly implicated in negative emotions and pain [90,91,92] emotional processes and the left ACC more involved in process positive emotions or happiness [93,94], which is consistent with the emotional lateralization model [95]. On these bases, our findings of different functional lateralization correlates involving the ACC may be linked to affective symptoms in LDD in a context of disrupted emotional and cognitive processing in affected patients. This seems to support the notion that indices of ACC dysfunction can aid in classifying subgroups of elderly patients with depressive disorders, demonstrating distinct illness courses and varying treatment needs [96]. Furthermore, such indices may prove useful for the development of new clinical applications and personalized treatments.

### 4.3. Striatum

Our meta-analysis revealed left sided hypoactivations of the putamen and caudate body in LLD compared to HCs. MDD adult patients with melancholic symptoms exhibited a smaller left putamen compared to non-melancholic subjects. Additionally, anhedonia symptoms correlated with both smaller left and right putamen volumes, although this volume loss may attenuate in older age [97]. Anhedonia has also been correlated with reduced grey matter volume in the left putamen and increased plasma interleukin-6 levels [98]. The neural functioning of the left putamen has also been implicated in the risk of bipolar disorder onset, suggesting a potential marker of vulnerability [99], as well as suicidality in bipolar II disorder [100]. Our results are consistent with decreased putamen regional homogeneity reported in adult patients with MDD [101,102], and with a recent SPECT study that showed negative correlation between levels of depression, anxiety, anhedonia and psychomotor retardation and DAT availability in the left putamen [103]. The same study showed lower left putamen DAT availability in seriously depressed patients and in patients with significant psychomotor retardation [103].

Regarding elderly patients, the left putamen volumes of LLD subjects expressing the COMT Met/Met genotype were smaller that the control subjects, which indicates that genetic and neural changes of this area could be considered as centrally involved in the etiopathogenesis of LLD [104]. Dysfunctions of the left putamen impact on movement preparation and execution in elderly patients with bipolar disorder depressive episode and motor retardation [105].

According to our results, the functional changes of the left putamen could negatively impact both salience and reward network functioning also in LLD patients. Furthermore, white matter abnormalities of the left putamen were reported in MDD treatment-naïve adult patients [106], which could be linked with the other structural and functional changes in the same area.

Regarding the left caudate hypoactivation we observed in LLD, this region has been associated with psychomotor dysfunction linked to reduced volume in LLD [107], suggesting a crucial involvement of the caudate nucleus in the motor aspects of depression during the elderly. The left caudate resting-state function has been also involved in first-episode treatment-naive LLD [19] and in late-life suicidality among LLD patients [108].

Functional alterations within some of the reward-related brain regions, such as the putamen and caudate body, underscore the intricate interplay between neural circuitry and depressive symptomatology in LLD. Specifically, the manifestation of anxiety, anhedonia and melancholic features, as well as motor symptoms, may reflect dysregulated reward processing mechanisms, implicating these regions as potential targets for understanding and treating LLD. Further clarifying the neurobiological correlates of reward dysfunction holds promise for advancing both diagnostic precision and therapeutic interventions in LLD.

### 4.4. Cerebellum

We found that LLD patients compared to healthy controls showed hyperactivation of the cerebellar right culmen and left declive.

The cerebellar declive is a region primarily involved in a wide range of functions, primarily including motor coordination and balance, but also encompassing cognitive processing and emotional regulation [109]. Specifically, the left declive has been implicated in spatial working memory and the accuracy of saccadic eye movements [110]. Our findings regarding cerebellar dysfunctions in LLD can be associated with existing evidence indicating the involvement of cerebellar vermis functional connectivity in the treatment response to serotonergic antidepressants in LLD [111]. Additionally, dysfunctional vermis connectivity in the posterior default mode network has been specifically linked to the neuropathophysiology of LLD [112]. Dysfunctional coupling between the cerebellum and ventromedial prefrontal cortex has been associated with cognitive symptoms, while cerebellum-posterior cingulate coupling has been linked to changes in emotional processing in LDD [113].

These findings can also be associated with consistent evidence of severe depressive symptom burden associated with smaller grey matter volumes of the cerebellum [114]. On these bases, it is important to clarify the differences between the neuroanatomical and neurofunctional correlates of aging on cerebellar circuits and those concerning LLD, also in relation to their impact on specific motor, cognitive, and affective operations, with the aim of developing procedures and clinical applications aimed at preventing the onset of depression and improving motor, cognitive, and affective symptoms in affected patients.

Taken together, our results can also be linked to existing evidence regarding decreased intrinsic brain functional connectivity and disrupted brain network topology in LLD, as reflected by alterations in both global and nodal network metrics [115], and that the neurofunctional changes of MDD evolve over the life course [116].

*Limits:* One limitation of the present study pertains to its epistemological nature, as it is arguable whether neural functional changes depicted with neuroimaging techniques fundamentally reflect the pathophysiology of LLD. Another limitation concerns the low number of included studies, attributed to the scarcity of research on this matter. Additionally, there is a methodological limitation in the aggregation of different tasks analysing diverse cognitive functions or the resting state. However, this aggregation allowed for a comprehensive exploration of neural functions, albeit at the cost of finer precision. Furthermore, there is a potential limitation in grouping medication-free patients with those on medication, where medication could have been a confounder across studies, especially in the context of LLD. A final bias is that three studies [19,29,30] used samples with overlapping sociodemographic characteristics, which might somewhat influence the independence of the samples.

The present study highlights the existence of specific neurofunctional correlates for depression with onset after the age of 60. However, given the lack of a unified definition of this syndrome and the scarcity of evidence regarding the link between these correlates and psychopathological dimensions, further studies are needed before these findings can be translated into concrete clinical applications. To date, the potential role of fMRI in the diagnosis of LLD, particularly in the differential diagnosis from neurocognitive disorders in the elderly, represents a significant prospect in geriatrics, neurology, and psychiatry. The advancement of functional neuroimaging techniques and data interpretation methodologies, including those based on machine learning and artificial intelligence, could lead to the creation of new clinical applications that consider these neurobiological correlates, aiding in diagnosis, therapeutic interventions, and prognosis.

## 5. Conclusions

Our meta-analysis has substantiated our primary hypothesis concerning the alterations observed in various cortical-limbic-striatal brain areas in LLD compared to healthy individuals. Notably, our study highlights significant relation between LLD diagnosis and hyperfunctioning of the left STG, alongside hypofunctioning of the right medial/superior frontal gyrus. These cortical functional alterations have implications for the affective, cognitive, and motor symptoms associated with LLD.

Moreover, our investigation reveals the involvement of different areas within the language network, particularly the left Brodmann Areas 45 and 42, in the neuropathophysiology of LLD. Our findings align with existing evidence indicating dysfunctional bilateral ACC activity, which contributes to affective symptoms such as anxiety, apathy, and cognitive impairment.

Another significant aspect of our analysis pertains to the functional dysregulation observed in the left striatum, which appears to play a crucial role in LLD diagnosis and the manifestation of symptoms such as anxiety, anhedonia, melancholic features, motor abnormalities, and suicidality.

Finally, our results underscore the centrality of the cerebellum in LLD, particularly in cognitive and motor symptomatology, suggesting the need for further investigations into its role in this disorder. It will be crucial to design experiments aimed at better understanding the nature of the brain alterations observed. This understanding is essential for translating the knowledge gained into the clinical context of patients with LLD.

## Figures and Tables

**Table 1:** Main characteristics of the included studies.

Study	Technique and Task/Functional Metric	Participants *n* (m, f; Mean Age y, SD)	Main Findings
Bobb et al., 2012 [26]	fMRI, Stop Signal Task	LLD: 15 (3, 12; 60.7, 4.7) HCs: 13 (4, 9; 62, 5.3)	The LLD group exhibited greater activations within the left fronto–striatal–limbic circuitry.
Brassen et al., 2008 [27]	fMRI, emotional evaluation task	LLD: 13 (0, 13; 66.4, 6.1) HCs: 13 (0, 13; 66.4, 6.1)	The LLD group revealed greater activation of the vmPFC when processing positive words compared to negative words.
Brendel et al., 2016 [28]	PET	LLD: 21 (11, 10; 77, 6.9) HCs: 29 (17, 12; 75.4, 6.3)	A significant hypometabolism was observed in the fronto–temporal cortices and PCC in LLD patients.
Briceño et al., 2015 [17]	fMRI, Facial Emotion Perception Test	LLD: 26 (12, 14; 65, 7.9) HCs: 25 (12, 13; 68, 8.4)	Different patterns of frontal, limbic, and basal ganglia activation were observed based on gender, with older men showing hyperactivation and women exhibiting hypoactivation.
Chen et al., 2012 [29]	fMRI, ReHo	LLD: 15 (6, 9; 67.53, 6.12) HCs: 15 (6, 9; 64.9; 3.7)	The LLD group exhibited lower ReHo in the right praecuneus and higher ReHo in the left STG and left Crus I of the cerebellum.
Guo et al., 2013 [30]	fMRI, ALFF	LLD: 15 (6, 9; 67.53; 6.12) HCs: 15 (6, 9; 64.9, 3.7)	Patients exhibited lower ALFF in the bilateral superior frontal gyrus and higher ALFF in the left superior temporal gyrus.
Huang et al., 2019 [31]	fMRI, emotional Stroop task	LLD: 55 (17, 38; 66.36, 5.42) HCs: 40 (15, 25; 68.1, 5.3)	LLD patients exhibited hyperactivation in the right anterior insula, right ACC, right inferior frontal gyrus, bilateral fusiform gyrus, bilateral lingual gyrus, right supramarginal gyrus, left postcentral gyrus, left posterior insula, left middle temporal gyrus, and left culmen.
Lee et al., 2013 [32]	fMRI, one-back working memory task	LLD: 14 (11, 3; 65.1, 4.9) HCs: 14 (9, 5; 64.8, 4.2)	LLD exhibited enhanced activity in the left middle frontal and left parietal regions, along with reduced deactivation in several temporal regions and the left amygdala within the masks. Additionally, LLD activated additional neural nodes in the middle frontal and middle temporal regions outside the masks.
Liu et al., 2012 [19]	fMRI, Cohe-ReHo	LLD: 15 (6, 9; 67.53, 6.12) HCs: 15 (6, 9; 64.9, 3.7)	The LLD group showed decreased Cohe-ReHo in the left caudate nucleus, right ACC, left dorsolateral prefrontal cortex, right angular gyrus, bilateral medial prefrontal cortex, and right praecuneus. Increased Cohe-ReHo was observed in the left cerebellum posterior lobe, left superior temporal gyrus, bilateral supplementary motor area, and right postcentral gyrus.
Liu et al., 2022 [33]	fMRI, PerAF method.	LLD: 45 (15, 30; 67.04, 4.51) HCs: 34 (16, 18; 65.1, 4)	The LLD group exhibited decreased PerAF differences in both the bilateral superior frontal gyrus, orbital part, and the bilateral ACC.
Piani et al., 2022 [34]	fMRI, Go/No Go Task	LLD: 12 (7, 5; 66.5, 9.1) HCs: 12 (5, 7; 68.7, 12.3)	LDD patients showed elevated fMRI responses in the left praecuneus and the right lingual gyrus.
Rao et al., 2015 [35]	fMRI, Go/No Go Task	LLD: 20 (genders not specified; 66.8) HCs: 17 (genders not specified; 67.9)	Patients showed greater activation in the right precentral gyrus, right middle frontal gyrus, left middle occipital gyrus, amygdala, globus pallidus, bilateral fusiform gyrus, caudate, right anterior cingulate gyrus, parahippocampal gyrus, supramarginal gyri, cuneus, and mammillary body.
Respino et al., 2019 [36]	fMRI, ReHo and network homogeneity	LLD: 33 (11, 21; 72.2, 6.6) HCs: 43 (18, 25; 73.4, 6.5)	LLD patients exhibited increased ReHo in both the dorsal ACC bilaterally and the right middle temporal gyrus.
Smith et al., 2009 [20]	PET	LLD: 16 (6, 10; 65.3, 9.1) HCs: 13 (5, 8; 67.4, 7.4)	Among the LLD group, increased cerebral glucose metabolism was observed in the right and left superior frontal gyrus, as well as the posterior inferior parietal lobule.
Steffens et al., 2017 [37]	fMRI, ALFF	LLD: 52 (14, 38; 71.35, 7.58) HCs: 36 (9, 27; 74.3, 7.5)	LLD patients showed increased ALFFs in the middle temporal cortex, insula, fusiform gyrus, and cerebellum, and reduced ALFFs in the supplementary motor cortex, inferior parietal cortex, midcingulate, and PCC/praecuneus.
Takami et al., 2007 [38]	fMRI, Verbal fluency task	LLD: 20 (8, 12; 63, 8) HCs: 10 (4, 6; 67.6, 9.7)	LLD patients showed hyperactivation of the prefrontal cortex and ACC, and hypoactivation of the parietal and temporal cortices.
Wang et al., 2008 [39]	fMRI, emotional oddball task	LLD: 12 (5, 7; 69.1, 6) HCs: 20 (8, 12; 73.1, 5.3)	LLD correlated with decreased activation in the executive system regions, including the right middle frontal, cingulate and inferior parietal cortices.
Weisenbach et al., 2014 [40]	fMRI, word list-learning task	LLD: 24 (10, 14; 65.8, 8.2) HCs: 23 (13, 10; 67.9, 8.1)	The LLD group exhibited hypoactivation in the hippocampus, parahippocampal gyrus, insula, and cingulate, while demonstrating significantly higher activation in the inferior frontal gyrus.
Wu et al., 2020 [41]	fMRI, Event related design: evoked memories by photos	LLD: 16 (0, 16; 69, 8) HCs: 18 (0, 18; 69, 8)	LLD showed task-related decreased activations of the bilateral STG, left inferior frontal gyrus, right parahippocampal gyrus, left cingulate gyrus, right insular lobe, and bilateral cerebellum anterior lobe.

Table legend. ACC: anterior cingulate cortex; ALFF: amplitude of low frequency fluctuations; Cohe-ReHo: coherence-based regional homogeneity; fMRI: functional magnetic resonance imaging; HCs: healthy controls; LLD: late-life depression; PCC: posterior cingulate cortex; PET: positron emission tomography; PerAF: percent amplitude of fluctuation; ReHo: regional homogeneity; SD: standard deviation; STG: superior temporal gyrus; vmPFC: ventromedial prefrontal cortex; y: years.

**Table 2:** Between-group differences in brain neural functioning.

**Contrast: LLD > HCs**							
**Cluster #**	**x**	**y**	**z**	**ALE**	**P**	**Z**	**Label**
1	−60	−30	14	0.017	2.091 × 10^−6^	46.020.885	Left STG, BA 42 **
2	18	−50	−8	0.014	4.14 × 10^−5^	39.361.618	Right Cerebellum Culmen/Lingual gyrus *
3	6	38	8	0.013	5.212 × 10^−5^	38.805.122	Right ACC, BA 24 *
4	−48	20	16	0.013	6.311 × 10^−5^	38.337.176	Left Inferior Frontal Gyrus, BA 45 *
5	−38	−84	−22	0.012	9.346 × 10^−5^	37.360.582	Left Cerebellum Declive/Lateral Occipital Cortex *
**Contrast: LLD < HCs**							
**Cluster #**	**x**	**y**	**z**	**ALE**	**P**	**Z**	**Label (Nearest Gray Matter within 5 mm)**
1	4	32	44	0.017	2.175 × 10^−6^	45.939.293	Right Medial Frontal Gyrus, BA 8 **
2	14	46	12	0.015	9.261 × 10^−6^	4.282.004	Right Medial Frontal Gyrus, BA 9 *
3	−20	−10	48	0.013	2.789 × 10^−5^	4.029.988	Left Cingulate Gyrus, BA 24 *
4	−20	6	14	0.012	8.703 × 10^−5^	37.539.575	Left Putamen *
5	−18	4	16	0.012	8.883 × 10^−5^	3.748.818	Left Caudate Body *

* Significant for uncorrected *p* < 0.0001. ** Significant for cluster lever correction *p* < 0.05.

## Data Availability

Not applicable.

## References

[1] Nebes R.D., Pollock B.G., Houck P.R., Butters M.A., Mulsant B.H., Zmuda M.D., Reynolds C.F. (2003). Persistence of Cognitive Impairment in Geriatric Patients Following Antidepressant Treatment: A Randomized, Double-Blind Clinical Trial with Nortriptyline and Paroxetine. J. Psychiatr. Res..

[2] O’Brien J.T., Lloyd A., McKeith I., Gholkar A., Ferrier N. (2004). A Longitudinal Study of Hippocampal Volume, Cortisol Levels, and Cognition in Older Depressed Subjects. Am. J. Psychiatry.

[3] Jeuring H.W., Stek M.L., Huisman M., Oude Voshaar R.C., Naarding P., Collard R.M., van der Mast R.C., Kok R.M., Beekman A.T.F., Comijs H.C. (2018). A Six-Year Prospective Study of the Prognosis and Predictors in Patients with Late-Life Depression. Am. J. Geriatr. Psychiatry Off. J. Am. Assoc. Geriatr. Psychiatry.

[4] Riedel-Heller S.G., Luppa M. (2013). Depression in late life—What does epidemiology add?. Psychiatr. Prax..

[5] Grayson L., Thomas A. (2013). A systematic review comparing clinical features in early age at onset and late age at onset late-life depression. J. Aff. Disord..

[6] Szymkowicz S.M., Gerlach A.R., Homiack D., Taylor W.D. (2023). Biological factors influencing depression in later life: Role of aging processes and treatment implications. Transl. Psychiatry.

[7] Luppa M., König H.-H., Heider D., Leicht H., Motzek T., Schomerus G., Riedel-Heller S.G. (2013). Direct Costs Associated with Depressive Symptoms in Late Life: A 4.5-Year Prospective Study. Int. Psychogeriatr..

[8] da Silva J., Gonçalves-Pereira M., Xavier M., Mukaetova-Ladinska E.B. (2013). Affective Disorders and Risk of Developing Dementia: Systematic Review. Br. J. Psychiatry J. Ment. Sci..

[9] Diniz B.S., Butters M.A., Albert S.M., Dew M.A., Reynolds C.F. (2013). Late-Life Depression and Risk of Vascular Dementia and Alzheimer’s Disease: Systematic Review and Meta-Analysis of Community-Based Cohort Studies. Br. J. Psychiatry J. Ment. Sci..

[10] Takamiya A., Vande Casteele T., Koole M., De Winter F.-L., Bouckaert F., Van den Stock J., Sunaert S., Dupont P., Vandenberghe R., Van Laere K. (2021). Lower Regional Gray Matter Volume in the Absence of Higher Cortical Amyloid Burden in Late-Life Depression. Sci. Rep..

[11] Alexopoulos G.S. (2005). Depression in the Elderly. Lancet.

[12] Steffens D.C., Otey E., Alexopoulos G.S., Butters M.A., Cuthbert B., Ganguli M., Geda Y.E., Hendrie H.C., Krishnan R.R., Kumar A. (2006). Perspectives on Depression, Mild Cognitive Impairment, and Cognitive Decline. Arch. Gen. Psychiatry.

[13] Maier A., Riedel-Heller S.G., Pabst A., Luppa M. (2021). Risk Factors and Protective Factors of Depression in Older People 65+. A Systematic Review. PLoS ONE.

[14] Alexopoulos G.S. (2019). Mechanisms and treatment of late-life depression. Transl. Psychiatry.

[15] Saberi A., Mohammadi E., Zarei M., Eickhoff S.B., Tahmasian M. (2022). Structural and Functional Neuroimaging of Late-Life Depression: A Coordinate-Based Meta-Analysis. Brain Imaging Behav..

[16] Du M., Liu J., Chen Z., Huang X., Li J., Kuang W., Yang Y., Zhang W., Zhou D., Bi F. (2014). Brain Grey Matter Volume Alterations in Late-Life Depression. J. Psychiatry Neurosci..

[17] Briceño E.M., Rapport L.J., Kassel M.T., Bieliauskas L.A., Zubieta J.-K., Weisenbach S.L., Langenecker S.A. (2015). Age and Gender Modulate the Neural Circuitry Supporting Facial Emotion Processing in Adults with Major Depressive Disorder. Am. J. Geriatr. Psychiatry Off. J. Am. Assoc. Geriatr. Psychiatry.

[18] Dombrovski A.Y., Szanto K., Clark L., Reynolds C.F., Siegle G.J. (2013). Reward Signals, Attempted Suicide, and Impulsivity in Late-Life Depression. JAMA Psychiatry.

[19] Liu F., Hu M., Wang S., Guo W., Zhao J., Li J., Xun G., Long Z., Zhang J., Wang Y. (2012). Abnormal Regional Spontaneous Neural Activity in First-Episode, Treatment-Naive Patients with Late-Life Depression: A Resting-State fMRI Study. Prog. Neuropsychopharmacol. Biol. Psychiatry.

[20] Smith G.S., Kramer E., Ma Y., Kingsley P., Dhawan V., Chaly T., Eidelberg D. (2009). The Functional Neuroanatomy of Geriatric Depression. Int. J. Geriatr. Psychiatry.

[21] Yuan Y., Zhang Z., Bai F., Yu H., Shi Y., Qian Y., Liu W., You J., Zhang X., Liu Z. (2008). Abnormal Neural Activity in the Patients with Remitted Geriatric Depression: A Resting-State Functional Magnetic Resonance Imaging Study. J. Affect. Disord..

[22] Eickhoff S.B., Bzdok D., Laird A.R., Kurth F., Fox P.T. (2012). Activation Likelihood Estimation Meta-Analysis Revisited. NeuroImage.

[23] Laird A.R., Fox P.M., Price C.J., Glahn D.C., Uecker A.M., Lancaster J.L., Turkeltaub P.E., Kochunov P., Fox P.T. (2005). ALE Meta-Analysis: Controlling the False Discovery Rate and Performing Statistical Contrasts. Hum. Brain Mapp..

[24] Turkeltaub P.E., Eickhoff S.B., Laird A.R., Fox M., Wiener M., Fox P. (2012). Minimizing Within-Experiment and within-Group Effects in Activation Likelihood Estimation Meta-Analyses. Hum. Brain Mapp..

[25] Page M.J., McKenzie J.E., Bossuyt P.M., Boutron I., Hoffmann T.C., Mulrow C.D., Shamseer L., Tetzlaff J.M., Akl E.A., Brennan S.E. (2021). The PRISMA 2020 Statement: An Updated Guideline for Reporting Systematic Reviews. BMJ.

[26] Bobb D.S., Adinoff B., Laken S.J., McClintock S.M., Rubia K., Huang H., Husain M.M., Mapes K.S., Tamminga C., Cullum C.M. (2012). Neural Correlates of Successful Response Inhibition in Unmedicated Patients with Late-Life Depression. Am. J. Geriatr. Psychiatry Off. J. Am. Assoc. Geriatr. Psychiatry.

[27] Brassen S., Kalisch R., Weber-Fahr W., Braus D.F., Büchel C. (2008). Ventromedial Prefrontal Cortex Processing during Emotional Evaluation in Late-Life Depression: A Longitudinal Functional Magnetic Resonance Imaging Study. Biol. Psychiatry.

[28] Brendel M., Reinisch V., Kalinowski E., Levin J., Delker A., Därr S., Pogarell O., Förster S., Bartenstein P., Rominger A. (2016). Hypometabolism in Brain of Cognitively Normal Patients with Depressive Symptoms Is Accompanied by Atrophy-Related Partial Volume Effects. Curr. Alzheimer Res..

[29] Chen J., Liu F., Xun G., Chen H., Hu M., Guo X., Xiao C., Wooderson S.C., Guo W., Zhao J. (2012). Early and Late Onset, First-Episode, Treatment-Naive Depression: Same Clinical Symptoms, Different Regional Neural Activities. J. Affect. Disord..

[30] Guo W., Liu F., Xun G., Hu M., Guo X., Xiao C., Chen H., Wooderson S.C., Chen J., Zhao J. (2013). Reversal Alterations of Amplitude of Low-Frequency Fluctuations in Early and Late Onset, First-Episode, Drug-Naive Depression. Prog. Neuropsychopharmacol. Biol. Psychiatry.

[31] Huang C.-M., Fan Y.-T., Lee S.-H., Liu H.-L., Chen Y.-L., Lin C., Lee T.M.C. (2019). Cognitive Reserve-Mediated Neural Modulation of Emotional Control and Regulation in People with Late-Life Depression. Soc. Cogn. Affect. Neurosci..

[32] Lee T.-W., Liu H.-L., Wai Y.-Y., Ko H.-J., Lee S.-H. (2013). Abnormal Neural Activity in Partially Remitted Late-Onset Depression: An fMRI Study of One-Back Working Memory Task. Psychiatry Res..

[33] Liu C., Pan W., Zhu D., Mao P., Ren Y., Ma X. (2022). Altered Intrinsic Brain Activity in Patients with Late-Life Depression: A Resting-State Functional MRI Study. Front. Psychiatry.

[34] Piani M.C., Maggioni E., Delvecchio G., Ferro A., Gritti D., Pozzoli S.M., Fontana E., Enrico P., Cinnante C.M., Triulzi F.M. (2021). Sexual Dimorphism in the Brain Correlates of Adult-Onset Depression: A Pilot Structural and Functional 3T MRI Study. Front. Psychiatry.

[35] Rao J.A., Kassel M.T., Weldon A.L., Avery E.T., Briceno E.M., Mann M., Cornett B., Kales H.C., Zubieta J.-K., Welsh R.C. (2015). The Double Burden of Age and Major Depressive Disorder on the Cognitive Control Network. Psychol. Aging.

[36] Respino M., Hoptman M.J., Victoria L.W., Alexopoulos G.S., Solomonov N., Stein A.T., Coluccio M., Morimoto S.S., Blau C.J., Abreu L. (2020). Cognitive Control Network Homogeneity and Executive Functions in Late-Life Depression. Biol. Psychiatry Cogn. Neurosci. Neuroimaging.

[37] Steffens D.C., Wang L., Manning K.J., Pearlson G.D. (2017). Negative Affectivity, Aging, and Depression: Results from the Neurobiology of Late-Life Depression (NBOLD) Study. Am. J. Geriatr. Psychiatry Off. J. Am. Assoc. Geriatr. Psychiatry.

[38] Takami H., Okamoto Y., Yamashita H., Okada G., Yamawaki S. (2007). Attenuated Anterior Cingulate Activation during a Verbal Fluency Task in Elderly Patients with a History of Multiple-Episode Depression. Am. J. Geriatr. Psychiatry Off. J. Am. Assoc. Geriatr. Psychiatry.

[39] Wang L., Krishnan K.R., Steffens D.C., Potter G.G., Dolcos F., McCarthy G. (2008). Depressive State- and Disease-Related Alterations in Neural Responses to Affective and Executive Challenges in Geriatric Depression. Am. J. Psychiatry.

[40] Weisenbach S.L., Kassel M.T., Rao J., Weldon A.L., Avery E.T., Briceno E.M., Ajilore O., Mann M., Kales H.C., Welsh R.C. (2014). Differential Prefrontal and Subcortical Circuitry Engagement during Encoding of Semantically Related Words in Patients with Late-Life Depression. Int. J. Geriatr. Psychiatry.

[41] Wu D., Chen T., Huang X., Chen L., Yue Y., Yang H., Hu X., Gong Q. (2020). The Role of Old Photos in Reminiscence Therapy in Elderly Women with Depressive Symptoms: A Functional Magnetic Resonance Imaging Study. Biol. Res. Nurs..

[42] Anderson T.J., Jenkins I.H., Brooks D.J., Hawken M.B., Frackowiak R.S., Kennard C. (1994). Cortical Control of Saccades and Fixation in Man. A PET Study. Brain J. Neurol..

[43] Brown S., Martinez M.J., Parsons L.M. (2006). Music and Language Side by Side in the Brain: A PET Study of the Generation of Melodies and Sentences. Eur. J. Neurosci..

[44] Hall N.M., Gjedde A., Kupers R. (2008). Neural Mechanisms of Voluntary and Involuntary Recall: A PET Study. Behav. Brain Res..

[45] Pinto M., Pellegrino M., Lasaponara S., Scozia G., D’Onofrio M., Raffa G., Nigro S., Arnaud C.R., Tomaiuolo F., Doricchi F. (2021). Number Space Is Made by Response Space: Evidence from Left Spatial Neglect. Neuropsychologia.

[46] Schnell K., Heekeren K., Schnitker R., Daumann J., Weber J., Hesselmann V., Möller-Hartmann W., Thron A., Gouzoulis-Mayfrank E. (2007). An fMRI Approach to Particularize the Frontoparietal Network for Visuomotor Action Monitoring: Detection of Incongruence between Test Subjects’ Actions and Resulting Perceptions. NeuroImage.

[47] Smoski M.J., Keng S.-L., Ji J.L., Moore T., Minkel J., Dichter G.S. (2015). Neural Indicators of Emotion Regulation via Acceptance vs Reappraisal in Remitted Major Depressive Disorder. Soc. Cogn. Affect. Neurosci..

[48] Levy-Cooperman N., Burhan A.M., Rafi-Tari S., Kusano M., Ramirez J., Caldwell C., Black S.E. (2008). Frontal Lobe Hypoperfusion and Depressive Symptoms in Alzheimer Disease. J. Psychiatry Neurosci..

[49] Robert G., Le Jeune F., Dondaine T., Drapier S., Péron J., Lozachmeur C., Sauleau P., Houvenaghel J.-F., Travers D., Millet B. (2014). Apathy and Impaired Emotional Facial Recognition Networks Overlap in Parkinson’s Disease: A PET Study with Conjunction Analyses. J. Neurol. Neurosurg. Psychiatry.

[50] Upadhyay J., Silver A., Knaus T.A., Lindgren K.A., Ducros M., Kim D.-S., Tager-Flusberg H. (2008). Effective and Structural Connectivity in the Human Auditory Cortex. J. Neurosci. Off. J. Soc. Neurosci..

[51] Calvert G.A., Campbell R. (2003). Reading Speech from Still and Moving Faces: The Neural Substrates of Visible Speech. J. Cogn. Neurosci..

[52] Rizzolatti G., Fogassi L., Gallese V. (2001). Neurophysiological Mechanisms Underlying the Understanding and Imitation of Action. Nat. Rev. Neurosci..

[53] Liu W., Li H., Lin X., Li P., Zhu X., Su S., Shi J., Lu L., Deng J., Sun X. (2022). Blunted Superior Temporal Gyrus Activity to Negative Emotional Expression after Mindfulness-Based Cognitive Therapy for Late-Life Depression. Front. Aging Neurosci..

[54] Wang J., Lyu H., Chen J., Lin S., Zheng H., Li J., Kong F., Gao J., Yu H., Hu Y. (2020). Cortical Alterations Are Associated with Depression in Subcortical Vascular Mild Cognitive Impairment Revealed by Surface-Based Morphometry. J. Alzheimers Dis. JAD.

[55] Guo W., Sun X., Liu L., Xu Q., Wu R., Liu Z., Tan C., Chen H., Zhao J. (2011). Disrupted Regional Homogeneity in Treatment-Resistant Depression: A Resting-State fMRI Study. Prog. Neuropsychopharmacol. Biol. Psychiatry.

[56] Hagoort P. (2005). On Broca, Brain, and Binding: A New Framework. Trends Cogn. Sci..

[57] Kim H. (2011). Neural Activity That Predicts Subsequent Memory and Forgetting: A Meta-Analysis of 74 fMRI Studies. NeuroImage.

[58] Collette F., Van der Linden M., Delfiore G., Degueldre C., Luxen A., Salmon E. (2001). The Functional Anatomy of Inhibition Processes Investigated with the Hayling Task. NeuroImage.

[59] Menon V., Adleman N.E., White C.D., Glover G.H., Reiss A.L. (2001). Error-Related Brain Activation during a Go/NoGo Response Inhibition Task. Hum. Brain Mapp..

[60] Rizzolatti G., Fadiga L., Matelli M., Bettinardi V., Paulesu E., Perani D., Fazio F. (1996). Localization of Grasp Representations in Humans by PET: 1. Observation versus Execution. Exp. Brain Res..

[61] Steffens D.C., Wang L., Pearlson G.D. (2019). Functional Connectivity Predictors of Acute Depression Treatment Outcome. Int. Psychogeriatr..

[62] Fu J.H., Wong K., Mok V., Hu X., Xiong Y., Chen Y., Tang W.K., Chen X., Wong A., Chu W. (2010). Neuroimaging Predictors for Depressive Symptoms in Cerebral Small Vessel Disease. Int. J. Geriatr. Psychiatry.

[63] Derbyshire S.W., Jones A.K. (1998). Cerebral Responses to a Continual Tonic Pain Stimulus Measured Using Positron Emission Tomography. Pain.

[64] Fichtenholtz H.M., Dean H.L., Dillon D.G., Yamasaki H., McCarthy G., LaBar K.S. (2004). Emotion-Attention Network Interactions during a Visual Oddball Task. Brain Res. Cogn. Brain Res..

[65] Li Y., Zhang L., Zhang R., Xu T., Feng T. (2022). The Neural Basis Linking Achievement Motivation with Procrastination: Left Precuneus Connectivity with Right Anterior Cingulate Cortex. Pers. Soc. Psychol. Bull..

[66] Nathaniel-James D.A., Fletcher P., Frith C.D. (1997). The Functional Anatomy of Verbal Initiation and Suppression Using the Hayling Test. Neuropsychologia.

[67] Schäfer R., Popp K., Jörgens S., Lindenberg R., Franz M., Seitz R.J. (2007). Alexithymia-like Disorder in Right Anterior Cingulate Infarction. Neurocase.

[68] Woodward T.S., Ruff C.C., Ngan E.T.C. (2006). Short- and Long-Term Changes in Anterior Cingulate Activation during Resolution of Task-Set Competition. Brain Res..

[69] Pujol J., López A., Deus J., Cardoner N., Vallejo J., Capdevila A., Paus T. (2002). Anatomical Variability of the Anterior Cingulate Gyrus and Basic Dimensions of Human Personality. NeuroImage.

[70] Vogt B.A., Nimchinsky E.A., Vogt L.J., Hof P.R. (1995). Human Cingulate Cortex: Surface Features, Flat Maps, and Cytoarchitecture. J. Comp. Neurol..

[71] Smith G.S., Reynolds C.F., Pollock B., Derbyshire S., Nofzinger E., Dew M.A., Houck P.R., Milko D., Meltzer C.C., Kupfer D.J. (1999). Cerebral Glucose Metabolic Response to Combined Total Sleep Deprivation and Antidepressant Treatment in Geriatric Depression. Am. J. Psychiatry.

[72] Monkul E.S., Silva L.A.P., Narayana S., Peluso M.A.M., Zamarripa F., Nery F.G., Najt P., Li J., Lancaster J.L., Fox P.T. (2012). Abnormal Resting State Corticolimbic Blood Flow in Depressed Unmedicated Patients with Major Depression: A (15)O-H(2)O PET Study. Hum. Brain Mapp..

[73] Wang Y.-M., Yang Z.-Y. (2022). Aberrant Pattern of Cerebral Blood Flow in Patients with Major Depressive Disorder: A Meta-Analysis of Arterial Spin Labelling Studies. Psychiatry Res. Neuroimaging.

[74] Huang X., Lai S., Lu X., Wang Y., Zhang Y., Chen G., Chen P., Ye K., Duan M., Song K. (2023). Cognitive Dysfunction and Neurometabolic Alternations in Major Depressive Disorder with Gastrointestinal Symptoms. J. Affect. Disord..

[75] Matsuo K., Harada K., Fujita Y., Okamoto Y., Ota M., Narita H., Mwangi B., Gutierrez C.A., Okada G., Takamura M. (2019). Distinctive Neuroanatomical Substrates for Depression in Bipolar Disorder versus Major Depressive Disorder. Cereb. Cortex.

[76] Bae J.N., MacFall J.R., Krishnan K.R.R., Payne M.E., Steffens D.C., Taylor W.D. (2006). Dorsolateral Prefrontal Cortex and Anterior Cingulate Cortex White Matter Alterations in Late-Life Depression. Biol. Psychiatry.

[77] Kang Y., Shin D., Kim A., You S.-H., Kim B., Han K.-M., Ham B.-J. (2024). The Effect of Inflammation Markers on Cortical Thinning in Major Depressive Disorder: A Possible Mediator of Depression and Cortical Changes. J. Affect. Disord..

[78] Graff-Guerrero A., De la Fuente-Sandoval C., Camarena B., Gómez-Martin D., Apiquián R., Fresán A., Aguilar A., Méndez-Núñez J.C., Escalona-Huerta C., Drucker-Colín R. (2005). Frontal and Limbic Metabolic Differences in Subjects Selected According to Genetic Variation of the SLC6A4 Gene Polymorphism. NeuroImage.

[79] Drevets W.C., Price J.L., Simpson J.R., Todd R.D., Reich T., Vannier M., Raichle M.E. (1997). Subgenual Prefrontal Cortex Abnormalities in Mood Disorders. Nature.

[80] Mayberg H.S., Liotti M., Brannan S.K., McGinnis S., Mahurin R.K., Jerabek P.A., Silva J.A., Tekell J.L., Martin C.C., Lancaster J.L. (1999). Reciprocal Limbic-Cortical Function and Negative Mood: Converging PET Findings in Depression and Normal Sadness. Am. J. Psychiatry.

[81] Lu X., Lai S., Luo A., Huang X., Wang Y., Zhang Y., He J., Chen G., Zhong S., Jia Y. (2023). Biochemical Metabolism in the Anterior Cingulate Cortex and Cognitive Function in Major Depressive Disorder with or without Insomnia Syndrome. J. Affect. Disord..

[82] Song Y., Huang C., Zhong Y., Wang X., Tao G. (2022). Abnormal Reginal Homogeneity in Left Anterior Cingulum Cortex and Precentral Gyrus as a Potential Neuroimaging Biomarker for First-Episode Major Depressive Disorder. Front. Psychiatry.

[83] Batail J.M., Corouge I., Combès B., Conan C., Guillery-Sollier M., Vérin M., Sauleau P., Le Jeune F., Gauvrit J.Y., Robert G. (2023). Apathy in Depression: An Arterial Spin Labeling Perfusion MRI Study. J. Psychiatr. Res..

[84] Zhang Y., Cui X., Ou Y., Liu F., Li H., Chen J., Zhao J., Xie G., Guo W. (2021). Differentiating Melancholic and Non-Melancholic Major Depressive Disorder Using Fractional Amplitude of Low-Frequency Fluctuations. Front. Psychiatry.

[85] Miao H., Zhong S., Liu X., Lai S., He J., Zhu Y., Song Z., Chen P., Wang Y., Jia Y. (2022). Childhood Trauma History Is Linked to Abnormal Brain Metabolism of Non-Medicated Adult Patients with Major Depressive Disorder. J. Affect. Disord..

[86] Zheng H., Jia F., Guo G., Quan D., Li G., Wu H., Zhang B., Fan C., He X., Huang H. (2015). Abnormal Anterior Cingulate N-Acetylaspartate and Executive Functioning in Treatment-Resistant Depression After rTMS Therapy. Int. J. Neuropsychopharmacol..

[87] Frodl T., Jäger M., Born C., Ritter S., Kraft E., Zetzsche T., Bottlender R., Leinsinger G., Reiser M., Möller H.-J. (2008). Anterior Cingulate Cortex Does Not Differ between Patients with Major Depression and Healthy Controls, but Relatively Large Anterior Cingulate Cortex Predicts a Good Clinical Course. Psychiatry Res..

[88] Li Y., Jakary A., Gillung E., Eisendrath S., Nelson S.J., Mukherjee P., Luks T. (2016). Evaluating Metabolites in Patients with Major Depressive Disorder Who Received Mindfulness-Based Cognitive Therapy and Healthy Controls Using Short Echo MRSI at 7 Tesla. Magn. Reson. Mater. Phys. Biol. Med..

[89] de Asis J.M., Stern E., Alexopoulos G.S., Pan H., Van Gorp W., Blumberg H., Kalayam B., Eidelberg D., Kiosses D., Silbersweig D.A. (2001). Hippocampal and Anterior Cingulate Activation Deficits in Patients with Geriatric Depression. Am. J. Psychiatry.

[90] Brügger M., Ettlin D.A., Meier M., Keller T., Luechinger R., Barlow A., Palla S., Jäncke L., Lutz K. (2011). Taking Sides with Pain—Lateralization Aspects Related to Cerebral Processing of Dental Pain. Front. Hum. Neurosci..

[91] Del Casale A., Ferracuti S., Rapinesi C., De Rossi P., Angeletti G., Sani G., Kotzalidis G.D., Girardi P. (2015). Hypnosis and Pain Perception: An Activation Likelihood Estimation (ALE) Meta-Analysis of Functional Neuroimaging Studies. J. Physiol. Paris.

[92] Watanabe H., Fitting S., Hussain M.Z., Kononenko O., Iatsyshyna A., Yoshitake T., Kehr J., Alkass K., Druid H., Wadensten H. (2015). Asymmetry of the Endogenous Opioid System in the Human Anterior Cingulate: A Putative Molecular Basis for Lateralization of Emotions and Pain. Cereb. Cortex.

[93] Dolan R.J., Fletcher P., Morris J., Kapur N., Deakin J.F., Frith C.D. (1996). Neural Activation during Covert Processing of Positive Emotional Facial Expressions. NeuroImage.

[94] Phillips M.L., Bullmore E.T., Howard R., Woodruff P.W., Wright I.C., Williams S.C., Simmons A., Andrew C., Brammer M., David A.S. (1998). Investigation of Facial Recognition Memory and Happy and Sad Facial Expression Perception: An fMRI Study. Psychiatry Res..

[95] Craig A.D.B. (2005). Forebrain Emotional Asymmetry: A Neuroanatomical Basis?. Trends Cogn. Sci..

[96] Alexopoulos G.S., Gunning-Dixon F.M., Latoussakis V., Kanellopoulos D., Murphy C.F. (2008). Anterior Cingulate Dysfunction in Geriatric Depression. Int. J. Geriatr. Psychiatry.

[97] Sachs-Ericsson N.J., Hajcak G., Sheffler J.L., Stanley I.H., Selby E.A., Potter G.G., Steffens D.C. (2018). Putamen Volume Differences Among Older Adults: Depression Status, Melancholia, and Age. J. Geriatr. Psychiatry Neurol..

[98] Lu S., Wu C., Jia L., Fang Z., Lu J., Mou T., Hu S., He H., Huang M., Xu Y. (2022). Increased Plasma Levels of IL-6 Are Associated with Striatal Structural Atrophy in Major Depressive Disorder Patients with Anhedonia. Front. Psychiatry.

[99] Nimarko A.F., Fischer A.S., Hagan K.E., Gorelik A.J., Lu Y., Young C.J., Singh M.K. (2021). Neural Correlates of Positive Emotion Processing That Distinguish Healthy Youths at Familial Risk for Bipolar Versus Major Depressive Disorder. J. Am. Acad. Child Adolesc. Psychiatry.

[100] Marchand W.R., Lee J.N., Garn C., Thatcher J., Gale P., Kreitschitz S., Johnson S., Wood N. (2011). Striatal and Cortical Midline Activation and Connectivity Associated with Suicidal Ideation and Depression in Bipolar II Disorder. J. Affect. Disord..

[101] Wang Y., Li X., Yan H., Zhang Q., Ou Y., Wu W., Shangguan W., Chen W., Yu Y., Liang J. (2022). Multiple Examinations Indicated Associations between Abnormal Regional Homogeneity and Cognitive Dysfunction in Major Depressive Disorder. Front. Psychol..

[102] Yang X., Ma X., Li M., Liu Y., Zhang J., Huang B., Zhao L., Deng W., Li T., Ma X. (2015). Anatomical and Functional Brain Abnormalities in Unmedicated Major Depressive Disorder. Neuropsychiatr. Dis. Treat..

[103] D’Onofrio A.M., Pizzuto D.A., Batir R., Perrone E., Cocciolillo F., Cavallo F., Kotzalidis G.D., Simonetti A., d’Andrea G., Pettorruso M. (2024). Dopaminergic Dysfunction in the Left Putamen of Patients with Major Depressive Disorder. J. Affect. Disord..

[104] Pan C.-C., McQuoid D.R., Taylor W.D., Payne M.E., Ashley-Koch A., Steffens D.C. (2009). Association Analysis of the COMT/MTHFR Genes and Geriatric Depression: An MRI Study of the Putamen. Int. J. Geriatr. Psychiatry.

[105] Liberg B., Adler M., Jonsson T., Landén M., Rahm C., Wahlund L.-O., Kristoffersen-Wiberg M., Wahlund B. (2013). The Neural Correlates of Self-Paced Finger Tapping in Bipolar Depression with Motor Retardation. Acta Neuropsychiatr..

[106] Zeng L.-L., Liu L., Liu Y., Shen H., Li Y., Hu D. (2012). Antidepressant Treatment Normalizes White Matter Volume in Patients with Major Depression. PLoS ONE.

[107] Van Cauwenberge M.G.A., Bouckaert F., Vansteelandt K., Adamson C., De Winter F.L., Sienaert P., Van den Stock J., Dols A., Rhebergen D., Stek M.L. (2021). A Longitudinal Study of the Association between Basal Ganglia Volumes and Psychomotor Symptoms in Subjects with Late Life Depression Undergoing ECT. Transl. Psychiatry.

[108] Lin C., Huang C.-M., Chang W., Chang Y.-X., Liu H.-L., Ng S.-H., Lin H.-L., Lee T.M.-C., Lee S.-H., Wu S.-C. (2024). Predicting Suicidality in Late-Life Depression by 3D Convolutional Neural Network and Cross-Sample Entropy Analysis of Resting-State fMRI. Brain Behav..

[109] Park I.S., Lee N.J., Rhyu I.J. (2018). Roles of the Declive, Folium, and Tuber Cerebellar Vermian Lobules in Sportspeople. J. Clin. Neurol..

[110] Geier C.F., Garver K.E., Luna B. (2007). Circuitry Underlying Temporally Extended Spatial Working Memory. NeuroImage.

[111] Emam H., Steffens D.C., Pearlson G.D., Wang L. (2019). Increased Ventromedial Prefrontal Cortex Activity and Connectivity Predict Poor Sertraline Treatment Outcome in Late-Life Depression. Int. J. Geriatr. Psychiatry.

[112] Li W., Ward B.D., Xie C., Jones J.L., Antuono P.G., Li S.-J., Goveas J.S. (2015). Amygdala Network Dysfunction in Late-Life Depression Phenotypes: Relationships with Symptom Dimensions. J. Psychiatr. Res..

[113] Alalade E., Denny K., Potter G., Steffens D., Wang L. (2011). Altered Cerebellar-Cerebral Functional Connectivity in Geriatric Depression. PLoS ONE.

[114] Arleo A., Bareš M., Bernard J.A., Bogoian H.R., Bruchhage M.M.K., Bryant P., Carlson E.S., Chan C.C.H., Chen L.-K., Chung C.-P. (2024). Consensus Paper: Cerebellum and Ageing. Cerebellum.

[115] Tan W., Ouyang X., Huang D., Wu Z., Liu Z., He Z., Long Y., REST-meta-MDD Consortium (2023). Disrupted intrinsic functional brain network in patients with late-life depression: Evidence from a multi-site dataset. J. Affect. Disord..

[116] Long Y., Li X., Cao H., Zhang M., Lu B., Huang Y., Liu M., Xu M., Liu Z., Yan C. (2024). Common and distinct functional brain network abnormalities in adolescent, early-middle adult, and late adult major depressive disorders. Psychol. Med..

